# Clinical Outcomes Following Bifurcation Techniques for Percutaneous Coronary Intervention

**DOI:** 10.3390/jcm12185916

**Published:** 2023-09-12

**Authors:** Chayakrit Krittanawong, Hafeez Ul Hassan Virk, Yusuf Kamran Qadeer, Umer Irshad, Zhen Wang, Mahboob Alam, Samin Sharma

**Affiliations:** 1 Cardiology Division, NYU Langone Health and NYU School of Medicine, New York, NY 10016, USA; 2 Harrington Heart & Vascular Institute, University Hospitals Cleveland Medical Center, Case Western Reserve University, Cleveland, OH 44195, USA; 3 Section of Cardiology, Baylor College of Medicine, Texas Heart Institute, Houston, TX 77030, USA; 4 Department of Medicine, Rawalpindi Medical University, Rawalpindi 46000, Pakistan; 5 Robert D. and Patricia E. Kern Center for the Science of Health Care Delivery, Mayo Clinic, Rochester, MN 55905, USA; 6 Division of Health Care Policy and Research, Department of Health Sciences Research, Mayo Clinic, Rochester, MN 55905, USA; 7 Cardiac Catheterization Laboratory of the Cardiovascular Institute, Mount Sinai Hospital, New York, NY 10029, USA

## 1. Introduction 

Bifurcation lesions account for 20% of all percutaneous coronary interventions and represent a complex subset which are associated with lower procedural success and higher rates of restenosis. However, the optimal bifurcation technique remains inconclusive. The objective of this study was to systematically evaluate the bifurcation strategy comparing 2-stent strategy versus the provisional stenting strategy on outcomes.

## 2. Methods

We systematically searched Ovid MEDLINE, Ovid Embase, Ovid Cochrane Database of Systematic Reviews, Scopus, and Web of Science from database inception in 1966 through April 2023 for studies evaluating the strategy for bifurcation PCI. The search strategies included MeSH and Embase terms as well as keywords including bifurcation, coronary. Outcomes include target vessel revascularization, myocardial infarction (MI), all-cause mortality, cardiac mortality, major adverse effects, stent thrombosis. Two reviewers extracted data from each study (e.g., authors, year of publication, location, sample size, characteristics, endpoints) using a standard extraction form, and the data were then assessed by a separate group of reviewers. We used the Newcastle–Ottawa quality assessment scale for study quality valuation. Conflicts were resolved through consensus. We used the D-L random effects model. Any results stratified by sex were separated as 2 cohorts. We defined substantial heterogeneity between the studies if I^2^ was >50%. Stata version 11 (StataCorp LLC, College Station, TX, USA) was used for analyses.

## 3. Results

We analyzed 37 studies that followed 33,387 individuals who underwent bifurcation PCI with an average follow-up of 22 months. The specific endpoints that we followed were myocardial infarction (MI), all-cause mortality, cardiac mortality, major adverse effects, stent thrombosis, target vessel revascularization (TVR), and target lesion revascularization (TLR). 

**Figure 1:**
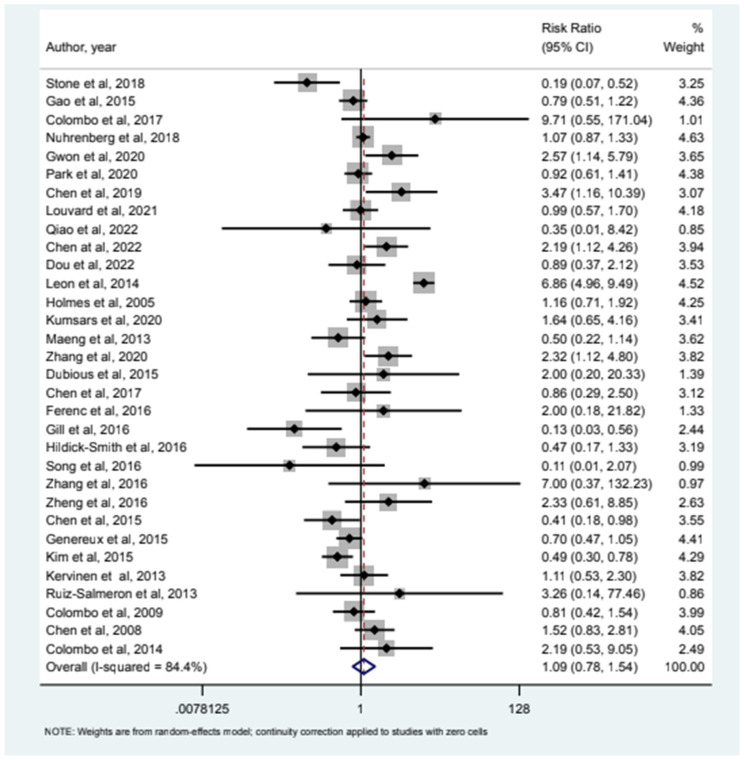
Forest plot and pooled risk ratio between bifurcation PCI and myocardial infarction.

**Figure 2:**
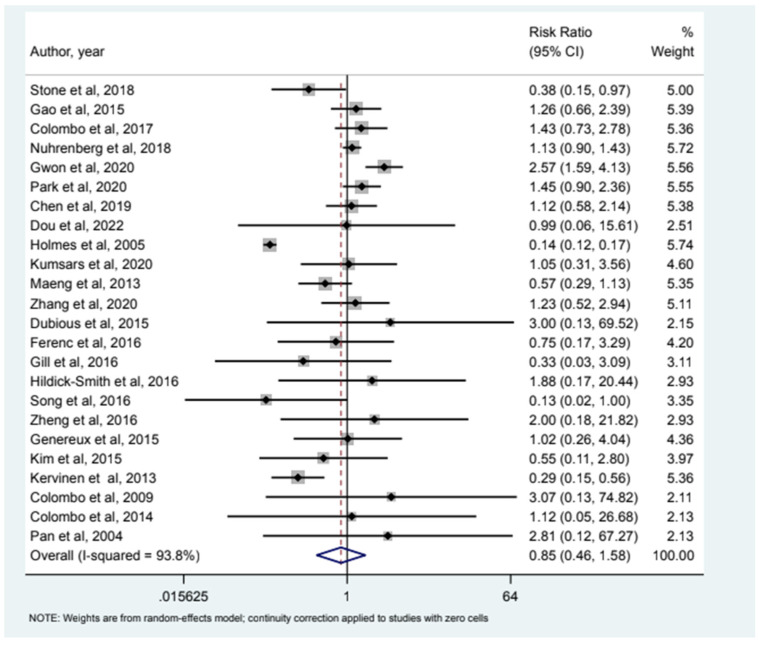
Forest plot and pooled risk ratio between bifurcation PCI and all-cause mortality.

**Figure 3:**
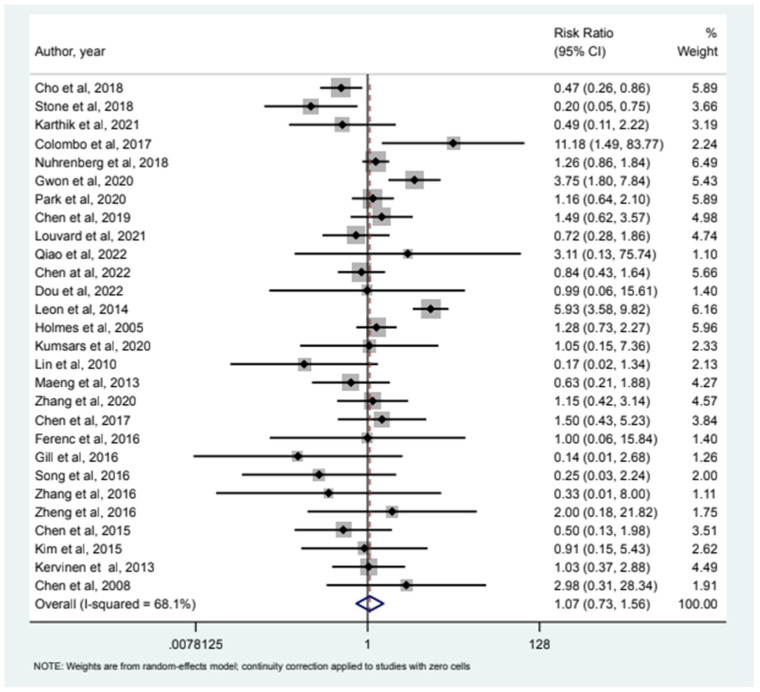
Forest plot and pooled risk ratio between bifurcation PCI and cardiac mortality.

**Figure 4:**
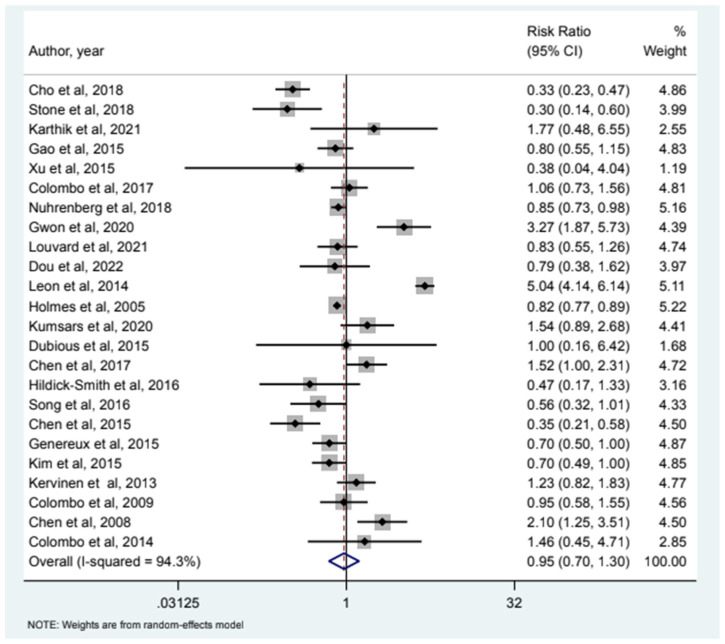
Forest plot and pooled risk ratio between bifurcation PCI and major adverse effects.

**Figure 5:**
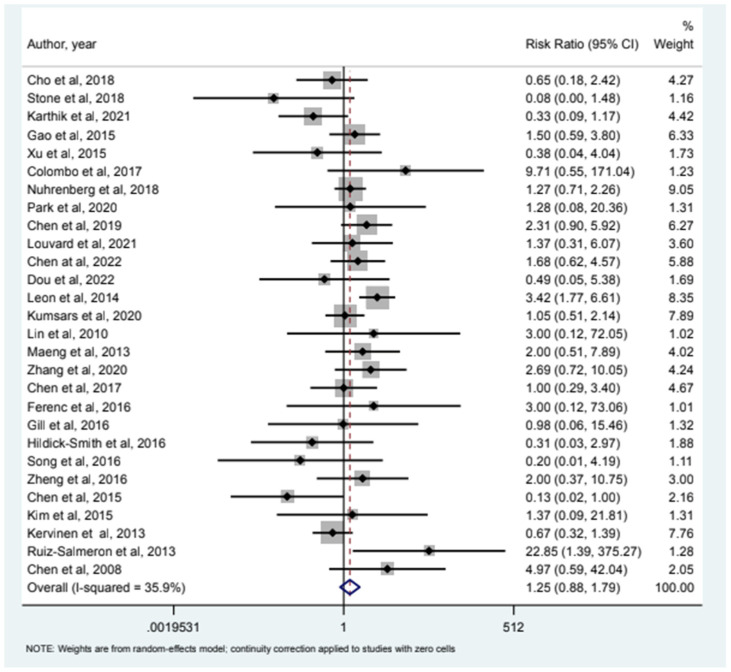
Forest plot and pooled risk ratio between bifurcation PCI and Stent thrombosis.

**Figure 6:**
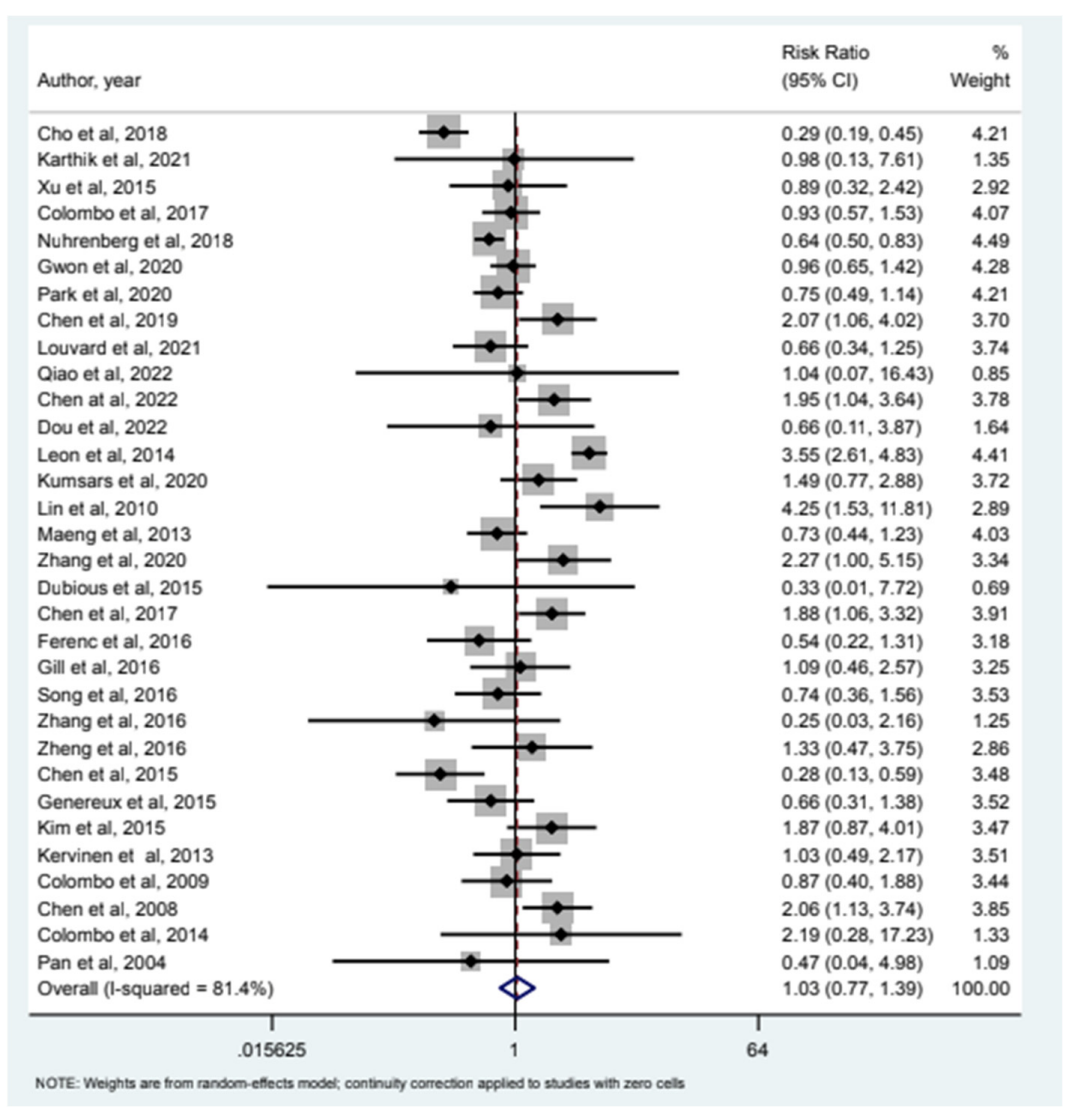
Forest plot and pooled risk ratio between bifurcation PCI and target risk revascularization.

**Figure 7:**
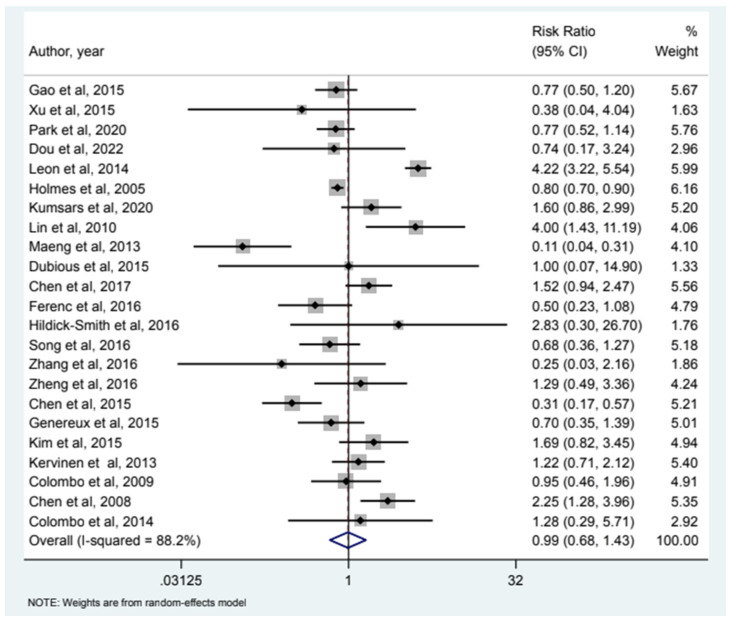
Forest plot and pooled risk ratio between bifurcation PCI and target vessel revascularization.

32 studies included MI as an endpoint and our analysis (see Figure 1) showed that relative risk of MI in the 2-stent strategy compared to the 1-stent strategy was 1.09 (95% CI (0.78–1.54)). 24 studies had all-cause mortality as an endpoint and the relative risk of all-cause mortality was 0.85 (95% CI (0.46–1.58)) as depicted in Figure 2. Cardiac Mortality was an endpoint in 28 studies and the relative risk of cardiac mortality (see Figure 3) was 1.07 (95% CI (0.73–1.56)). 24 studies listed major adverse effects as an endpoint and the relative risk of major adverse effects was 0.95 (95% CI (0.7–1.3)) as depicted in Figure 4. Stent thrombosis was analyzed in 28 studies (see Figure 5) and the relative risk of stent thrombosis was 1.25 (95% CI (0.88–1.79)). 32 studies evaluated target lesion revascularization and the relative risk of target risk revascularization was 1.03 (95% CI (0.77–1.39)) as seen in Figure 6. Finally, TVR was studied in 23 studies (see Figure 7) and the relative risk of TVR was 0.99 (95% CI (0.68–1.43)). Our results indicated that there was no significant difference regarding 2-stent strategy when compared to the traditional 1-stent strategy for bifurcation percutaneous intervention in regards to the endpoints of myocardial infarction (MI), all-cause mortality, cardiac mortality, major adverse effects, stent thrombosis, TVR, and TLR. 

## 4. Discussion

It has been established that bifurcation percutaneous coronary intervention compared with non-bifurcation percutaneous intervention is associated with a lower success rate, as well as a higher incidence of procedural and peri-procedural complications [1,2]. In addition, the single stent strategy is appealing because of it is faster with lower contrast use and radiation exposure. Given that bifurcation lesions make up 20% of all lesions treated with percutaneous coronary intervention, the optimal approach for approaching these lesions has been widely debated [3]. The first management decision is to decide whether to attempt a provisional stenting strategy or a 2-stent strategy. The European Bifurcation Club proposes that provisional stenting should be the preferred option for most bifurcation lesions, with the 2-stent strategy being reserved for bifurcation lesions that are more complex in nature and with a significant side branch supplying a large area of myocardium [4]. 

Our comprehensive meta-analysis compared the 2-stent strategy to the provisional stenting strategy for bifurcation lesions and found that there was no significant difference regarding the endpoints of myocardial infarction (MI), all-cause mortality, cardiac mortality, major adverse effects, stent thrombosis, TVR, and TLR. 

The DEFINITION-II Trial was a large multi-center, randomized trial that looked at 653 patients randomized to either a 2-stent technique or provisional stenting for bifurcation lesions. They found that after follow-up for one year, target lesion failure occurred in 11.4% of patients in the provisional stenting group compared to 6.1% in the 2-stent group (HR 0.52, 95% CI (0.3–0.9) with *p* value = 0.019), giving credence to the argument that 2-stent strategy may be beneficial to provisional stenting for bifurcation lesions [5]. It is worth noting though that 77.8% of the patients in the 2-stent strategy underwent the double kissing crush technique. Similarly, a study conducted by Ford and colleagues in 2018 also sought to evaluate the difference between provisional stenting and 2-stent strategy technique for bifurcation lesions. They found that while provisional stenting was associated with a reduction in all-cause mortality, that there was no difference in the endpoints of major adverse cardiac events, MI, stent thrombosis, or TLR [6]. Of note, the differences in all-cause mortality was not clearly elucidated, and factors such as stent thrombosis and duration of dual-antiplatelet therapy may have confounded the data.

Our current findings are corroborated by findings from other significant systematic reviews and meta-analyses. A meta-analysis conducted by Park and colleagues evaluated 29 randomized clinical trials to compare 2-stent strategy vs. provisional stenting for bifurcation lesions. They found that the two-stent strategy was not superior to provisional stenting except when the lesion length of the side branch is greater than 10 mm. In addition, of all the 2-stent techniques, they found that the double kissing crush technique was associated with lower risk of cardiac death, major adverse cardiac events, MI, stent thrombosis, TVR, and TLR [7]. Another meta-analysis conducted by Crimi and colleagues looked at 26 randomized clinical trials and found that the double kissing crush technique was associated with lower cardiac death, target vessel MI, stent thrombosis, TLR, and TVR [8]. Likewise, a meta-analysis conducted by Di Gioia and colleagues examined 5711 patients and found that the double crush kissing technique compared to provisional stenting had a lower risk of major cardiac adverse events, specifically driven by a reduction in TLR [9]. 

The greater role of double kissing crush technique was evaluated in the DKCRUSH-II trial which analyzed 370 patients with bifurcation lesions over 5 years. They found that the double kissing crush technique was associated with lower rates of TLR [10]. Likewise, the DKCRUSH-V trial randomized 482 patients with distal LM bifurcation to provisional stenting or double kissing crush technique. They found that after 1 year that the double kissing crush technique resulted in lower rates of target lesion failure when compared to provisional stenting [11]. It is worth noting that this study specifically enrolled patients with left main coronary artery disease, and with significant side branch involvement. Thus, it may seem that the more complex the bifurcation lesion is, the greater success that double kissing technique may have. 

There are several explanations as to why the double kissing crush technique may be the most advantageous of all the two-stent techniques. One mechanism is that the technique is not influenced by the bifurcation angle and maintains the wire access in the main vessel [12]. There is also some evidence to suggest that the double kissing crush technique utilizes the final kissing balloon inflation more often, which results in improved stent apposition, stent geometry, and reductions in flow disturbance [13]. 

Our study has limitations. The first limitation is that every study we analyzed varied in the complexity of lesions, such as left main involvement, type of bifurcation (medina classification), and extent of lesion in the side branch. Similarly, large bifurcation lesions (side branch > 2.5 mm–4 mm) are underrepresented in these trials so this analysis may not be applicable to all the bifurcation PCIs. Another limitation is the use of advanced imaging such as intravascular ultrasound or dedicated CT imaging to help operators plan their intervention. Finally, skill of operators as well as utilization of type of dual-antiplatelet therapy as well as duration varied as well. 

Our findings corroborate that 2-stent strategy vs. provisional stenting for bifurcation lesions does not result in statistically significant differences when compared to the endpoints of myocardial infarction (MI), all-cause mortality, cardiac mortality, major adverse effects, stent thrombosis, TVR, and TLR. This makes the argument for utilizing provisional stenting for less complex bifurcation lesions, as we have found sufficient evidence to support the utilization of 2-stent techniques (specifically double kissing crush technique) for bifurcations with more complex anatomy and side branch involvement. As more research continues to be developed, more guidelines need to be developed addressing questions such as the use of intravascular ultrasound and optimal anti-platelet therapy in bifurcation lesions, as these could potentially affect patient outcomes.
